# Acupuncture in the treatment of inflammation-related ocular degenerations: a systematic review

**DOI:** 10.3389/fmed.2026.1749297

**Published:** 2026-03-25

**Authors:** Mario Alberto Bautista-Hernández, Rafael Torres-Rosas, Liliana Argueta-Figueroa, Adriana Moreno-Rodríguez, Yobana Pérez-Cervera, Alfonso Enrique Acevedo-Mascarúa, Enrique Antonio Martínez-Martínez

**Affiliations:** 1 Doctorado en Biociencias (SNP 005846), Facultad de Medicina Universidad Autónoma “Benito Juárez” de Oaxaca, UABJO, Oaxaca, Mexico; 2 Departamento de Ortodoncia, Centro de Estudios en Ciencias de la Salud y la Enfermedad, Facultad de Odontología, Universidad Autónoma “Benito Juárez” de Oaxaca, UABJO, Oaxaca, Mexico; 3 Laboratorio de Medicina Complementaria, Centro de Estudios en Ciencias de la Salud y la Enfermedad, Facultad de Odontología, Universidad Autónoma “Benito Juárez” de Oaxaca, UABJO, Oaxaca, Mexico; 4 Tecnológico Nacional de México/Instituto Tecnológico de Toluca, Metepec, Mexico; 5 Secretaria de Ciencia, Humanidades, Tecnologia e Innovacion, Mexico City, Mexico; 6 Laboratorio de Estudios Epidemiológicos, Clínicos, Diseños Experimentales e Investigación, Facultad de Ciencias Químicas, Universidad Autónoma “Benito Juárez” de Oaxaca, UABJO, Oaxaca, Mexico

**Keywords:** complementary medicine, diabetic retinopathy, dry eye syndrome, glaucoma, inflammation, macular degeneration

## Abstract

**Background:**

Inflammation-related ocular degenerations include dry eye syndrome, diabetic retinopathy, age-related macular degeneration, and glaucoma. Until now, there are no efficient protocols for treating inflammation-related ocular degenerations. On the other hand, acupuncture therapy modulates the inflammatory and oxidative stress responses in several inflammatory diseases and has been proposed as an emerging therapy for the potential treatment of ocular pathologies.

**Objective:**

This work aimed to conduct a comprehensive systematic review to address the efficacy of acupuncture therapy for Inflammation-related ocular degenerations.

**Methods:**

The designs of the included studies were clinical trials and observational studies, whereas case series, *in vivo,* and *in vitro* studies were excluded. The search was performed in five databases. Relevant data from all included studies were recorded, the outcomes analyzed were intraocular pressure and visual acuity for glaucoma; score of symptoms, pain, tear-film breakup time, and mm of Schirmer test for dry eye syndrome; visual acuity, retinal structure or disease progression for diabetic retinopathy; and finally visual acuity and scores of symptoms for age-related macular degeneration. Risk of bias (using the RoB 2 tool) and quality (using the Grading of Recommendations Assessment, Development, and Evaluation [GRADE] tool) assessments were conducted.

**Results:**

The included studies showed heterogeneity, risk of bias, inconsistency, and very low certainty of evidence.

**Conclusion:**

There is no conclusive evidence to support that acupuncture is an effective therapy in patients with inflammation-related ocular degeneration disease.

**Systematic Review Registration:**

https://www.crd.york.ac.uk/PROSPERO/view/CRD42022368882, identifier PROSPERO (CRD42022368882).

## 1 Introduction

Inflammation-related ocular degenerations include a heterogeneous group of disorders such as dry eye syndrome, diabetic retinopathy (DR), age-related macular degeneration (AMD), and glaucoma. Chronic inflammation is a fundamental component of the onset, progression, and outcome of these disorders, even though the initial drive is not immune-related (1). However, the research concerning the immune system’s role in ocular degenerative diseases (ODD) is growing. Nevertheless, elucidating the inflammatory pathways implicated in ODD’s pathogenesis is required to develop efficient immunotherapies (2).

Oxidative stress plays a central role in inflammation-related ocular degeneration; the key oxidative mechanisms driving these diseases include excess reactive oxygen species (ROS), mitochondrial dysfunction, and chronic inflammation (3). These mechanisms are related to ocular characteristics; for example, the eye is highly exposed to light, oxygen, and metabolic activity, making it vulnerable to oxidative damage. Also, ultraviolet radiation, pollution, and hyperglycemia contribute to ROS overproduction (4). Nevertheless, retinal cells require high energy, making mitochondria critical and leading to mitochondrial damage, electron leakage, and increased ROS production (5). Finally, ROS activates NF-κB, a key transcription factor that promotes inflammation; this leads to the release of pro-inflammatory cytokines and the development of chronic inflammation, exacerbating oxidative damage and creating a vicious cycle (6).

Neuroendocrine immunomodulation represents the main mechanism of acupuncture (7), as exemplified by the fact that acupuncture attenuates inflammation by modulating neurotransmitters and hormones, such as catecholamines, acetylcholine, neuropeptides, and cortisol (7–10). Acupuncture therapy modulates the inflammatory response in several animal disease models (11–13). In addition, acupuncture treatment reduces the release of pro-inflammatory cytokines in humans (14–16). Consequently, this therapy reduces the inflammatory process, relieving the discomfort in local and systemic inflammatory diseases.

Acupuncture has been reported to increase the activity of antioxidant enzymes, such as superoxide dismutase, catalase, and glutathione peroxidase. Nevertheless, acupuncture regulation of cortisol affects oxidative stress levels and diminishes cytokine production, which plays a role in oxidative damage (17). However, there are still many inflammatory diseases in which the effectiveness of acupuncture in modulating the inflammatory process has not been proven.

There are no effective protocols for treating ODD, and to the best of our knowledge, there is no specific pharmacological treatment for these conditions. Also, there is no FDA-approved immunotherapy for ODD. Due to the above, this study aims to perform a systematic review of acupuncture as a therapy to modulate inflammation and reduce symptoms of ODD.

## 2 Methodology

This study was performed according to the Preferred Reporting Items for Systematic Reviews and Meta-Analyses (PRISMA) (18) and Cochrane guidelines (19).

### 2.1 Protocol registry

The protocol was registered (CRD42022368882) in the International Prospective Register of Systematic Reviews (PROSPERO).

### 2.2 Study design

The types of included studies in the systematic review were randomized clinical trials and observational studies. On the other hand, case reports, case series, letters, comments, short communications, pilot studies (n < 10), animal studies, *in vitro* studies, and literature reviews were excluded.

### 2.3 Eligibility criteria and participant characteristics of studies

The eligibility criteria of the included studies were defined considering the PICO (Population, Intervention, Comparator, and Outcome) model as follows:

#### 2.3.1 Population

The population must be patients with ODD (diabetic retinopathy, dry eye syndrome, age-related macular degeneration, or glaucoma).

#### 2.3.2 Intervention

The intervention involved acupuncture on corporeal points or microsystems such as the hand, the foot, and the ear. The intervention must be performed with traditional acupuncture placement (needle puncture with manual stimulation or electroacupuncture). On the other hand, moxibustion, laser applied at acupuncture points, and fire needles, among others, were excluded.

#### 2.3.3 Comparator

The included studies should incorporate standard care or a placebo (sham acupuncture) as the control group.

The standard care for DR may include:

For early stages (mild/moderate non-proliferative DR), regular dilated retinal examinations and optimized systemic management. Upon progression to severe non-proliferative DR (NPDR) or high-risk proliferative DR (PDR), timely panretinal photocoagulation (PRP) laser therapy, though intravitreal anti-vascular endothelial growth factor (anti-VEGF) injections (e.g., aflibercept, ranibizumab, bevacizumab) (20).

The standard care for dry eye syndrome (DES) may include:

First-line therapy such as patient education on environmental modifications (e.g., reducing screen time, humidifiers, hydration) and consistent use of preservative-free artificial tear substitutes for lubrication. Eyelid hygiene (warm compresses, lid massage, cleansing wipes) for evaporative dry eye associated with MGD.

For persistent moderate-to-severe DES topical prescription anti-inflammatory agents such as calcineurin inhibitors (e.g., cyclosporine 0.05–0.1%, lifitegrast 5%). Oral omega-3 fatty acid supplementation.

The standard care for age-related macular degeneration may include:

First-line therapy such as patient education of the risk factor modification (smoking cessation, UV protection, AREDS-2 formula supplements for intermediate dry AMD in appropriate patients), regular monitoring.

For dry AMD, nutritional supplementation (vitamins C, E, lutein, zeaxanthin, zinc, copper based on AREDS-2 criteria) and lifestyle counseling, complement inhibitors (e.g., pegcetacoplan, avacincaptad pegol) for slowing progression of geographic atrophy (GA).

For wet AMD, intravitreal anti-VEGF therapy (e.g., aflibercept, ranibizumab, brolucizumab, faricimab), and laser photocoagulation.

Standard care for glaucoma may include:

Pharmacotherapy such as prostaglandin analogs (e.g., latanoprost, travoprost, bimatoprost) or combination drops (e.g., beta-blockers, alpha-agonists, carbonic anhydrase inhibitors).

#### 2.3.4 Outcomes

The main outcomes are, according to the ODD, as follows:

Glaucoma: intraocular pressure (IOP) and visual acuity.

Dry eye syndrome: score of symptoms, pain, tear-film breakup time (BUT) for tear-film instability, and mm of Schirmer test (S1T).

Diabetic retinopathy: visual acuity, retinal structure or disease progression.

Age-macular degeneration: visual acuity and scores of symptoms (blurred vision, visual fatigue, functional reading independence index score).

### 2.4 Search strategy and databases used

The algorithms used for the search strategy are shown in Table 1. Two reviewers (PCY and AEAM) searched five electronic databases: PubMed, ClinicalTrials.gov, Web of Science, Scopus, and Science Direct. The manual search was achieved by examining the references from the studies included in the review. Each database was searched from the start date until February 2025.

**Table 1:** Keywords and algorithms used in the search strategy.

Focused question	Is there evidence that acupuncture treatment is an effective therapy for symptom relief in patients with inflammation-related to ocular degenerations disease?
Keywords	Dry eye, xerophthalmia, Sjögren’s syndrome, keratoconjunctivitis Sicca Glaucoma, ocular hypertension Macular degeneration, maculopathy, maculopathies, macular dystrophy Diabetic retinopathy, diabetic Retinopathies Acupuncture, electroacupuncture
Algorithms	
Pubmed	(“diabetic retinopathy” OR “dry eye” OR “xerophthalmia” OR “Sjögren’s syndrome” OR “Keratoconjunctivitis Sicca” OR “macular degeneration” OR “maculopathy” OR Maculopathies OR “Macular Dystrophy” OR glaucoma OR “ocular hypertension”) AND (acupuncture OR electroacupuncture)
Clinical Trials	Filters: Completed Studies | Studies With Results | Interventional Studies | Condition or disease: “diabetic retinopathy” OR “dry eye” OR “xerophthalmia” OR “Sjögren’s syndrome” OR “Keratoconjunctivitis Sicca” OR “macular degeneration” OR maculopathy OR glaucoma OR “ocular hypertension” Intervention/Treatment: acupuncture OR electroacupuncture
Web of Science	(ALL = (“diabetic retinopathy” OR “dry eye” OR “xerophthalmia” OR “Sjögren’s syndrome” OR “Keratoconjunctivitis Sicca” OR “macular degeneration” OR maculopathy OR glaucoma OR “ocular hypertension”)) AND ALL = (acupuncture OR electroacupuncture)
Scopus	TITLE-ABS-KEY ((“diabetic retinopathy” OR “dry eye” OR “xerophthalmia” OR “Sjögren’s syndrome” OR “Keratoconjunctivitis Sicca” OR “macular degeneration” OR maculopathy OR glaucoma OR “ocular hypertension”) AND (acupuncture OR electroacupuncture))
Science Direct	(“diabetic retinopathy” OR “dry eye” OR “xerophthalmia” OR “Sjögren’s syndrome” OR “Keratoconjunctivitis Sicca” OR “macular degeneration” OR “maculopathy” OR glaucoma OR “ocular hypertension”) AND (acupuncture OR electroacupuncture)

### 2.5 Study selection

The selection studies process was carried out by two reviewers (MRA and BHMA). A third reviewer (RTR) resolved disagreements. The eligibility of the studies that could be included in the review was determined by reading the title and summary of each record identified in the search.

### 2.6 Data collection process and data items

A standardized Microsoft Excel (Microsoft Corp., Redmond, USA) worksheet was prepared to register the relevant information and outcomes of all the studies included in the systematic review, such as participant demographics and baseline characteristics, methodology, number of sessions and frequency, time intervals assessments, and the outcomes results. The outcomes data were expressed as the mean and standard deviation values at baseline and the longest reported follow-up interval. Two reviewers (BHMA and YPC) were responsible for data extraction. A third reviewer (AFL) resolved disagreements. The study researchers were contacted via email for information concerning missing data or additional details without response (21, 22).

### 2.7 Risk of bias in individual studies and quality assessment

The risk of bias assessment process was carried out by two reviewers (EAMM and AMR). A third reviewer (RTR) resolved disagreements. For assessment of the risk of bias of the studies included in the review, the recommendations from the Cochrane Handbook for Systematic Reviews of Interventions Chapter 8 (23) and Risk of Bias 2 (RoB 2) (24), or The Newcastle-Ottawa quality assessment scale (NOS) (25) tools were used for randomized clinical trials and observational studies, respectively. The figure of the risk of bias assessment was built as in previous research (26). Additionally, we evaluated the quality of each study using the Grading of Recommendations Assessment, Development, and Evaluation (GRADE) criteria.

## 3 Results

### 3.1 Selection and characteristics of the studies

**Figure 1:**
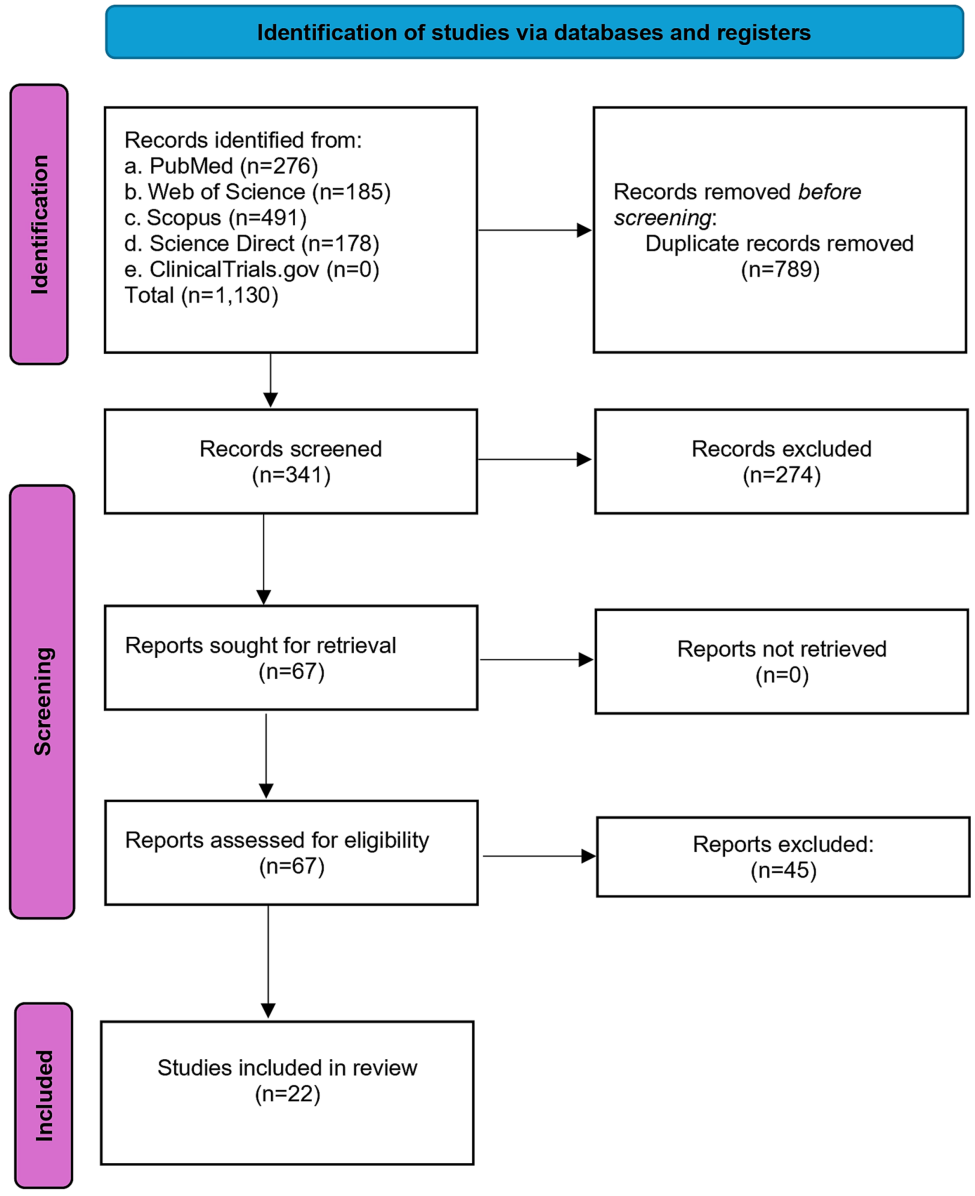
PRISMA diagram flow for the selection of the included studies.

The initial search yielded 1,130 records. Subsequently, 789 duplicate records were eliminated, leaving 341 records reviewed by title and abstract. 58 full-text studies were reviewed, but only 22 met the eligibility criteria, as shown in Figure 1. On the other hand, the studies that did not fully meet the eligibility criteria are shown in Appendix A1.

**Figure 2:**
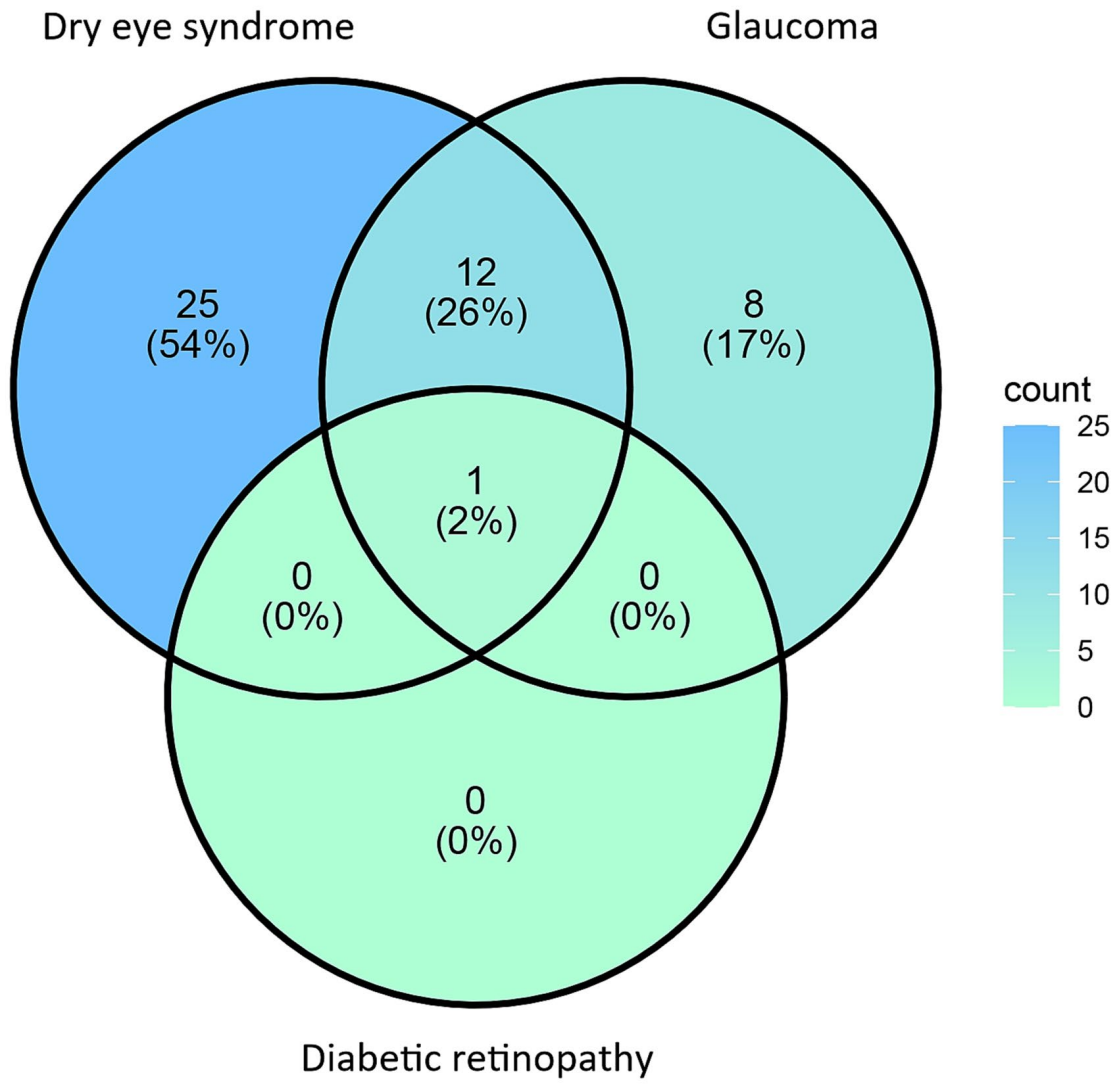
Relationship between the acupuncture points and Inflammation-related ocular degeneration treatments.

A total of 46 acupuncture points have been reported in the treatment of inflammation-related ocular degenerations: 38 for dry eye syndrome, 21 for glaucoma, and one for diabetic retinopathy. Additionally, 28% of these acupuncture points are commonly used in treatments for dry eye syndrome and glaucoma, as shown in Table 2 and Figure 2. Of the 22 studies reviewed, 13 did not report any information regarding the background or qualifications of the acupuncture practitioners. Additionally, 15 studies lacked sufficient detail about the treatment protocols. Regarding the treatment regimen, the average number of acupuncture sessions was 20, with each session lasting 20 min.

**Table 2:** Summary of characteristics of the interventions.

ID	Acupoint and practitioner background	Treatment details	Treatment regimen
Dry eye syndrome			
Neep J, et al. (1998) (33)	GB1, UB2, ST5, EXHN3, LI4, SI3, LI3, KI6, SJ5 No data concerning the practitioner’s background	Needle: No data Deep insertion: No data Stimulation: No data	30 min per session Once per week for ten weeks
Grönlund MA, et al. (2004) (21)	ST2, ST8, ST36, GB1, GB14, BL2, LI4 No data concerning the practitioner’s background	Needle: Placed bilateral. Deep insertion: No data	30 min per session Once to twice per week. A total of ten sessions.
Tseng KL, et al. (2006) (34)	EXHN5, SJ23, GB14, ST2, SP6. No data concerning the practitioner’s background.	Needle: No.36 to face, No.32 for extremities stimulated on both sides. Deep insertion: No data Electroacupuncture stimulation: 12 mA in face, 20 mA on the legs. Frequency 100 Hz and 15 Hz in successive 10 s intervals.	20 min per session. Two times per week for eight weeks.
Shin MS, et al. (2010) (36)	GV23, BL2, GB14, SJ23, EXHN3, ST1, GB20, SP3, LU9, LU10, HT8. Acupuncture was offered by licensed oriental medicine physicians with at least 5 years of clinical experience in acupuncture treatment.	Needle: No data Deep insertion: No data	20 min per session Three times per week for three weeks.
Gong L, et al. (2010) (35)	BL1, BL2, GB14, SJ23, ST2, LI4, LR3, GB37, SP6, GB20, EXHN5 No data concerning the practitioner’s background.	Needle: No data Deep insertion: No data	20 min per session. Three times per week for 21 weeks.
Kim TH, et al. (2012) (22)	BL2, GB14, SJ23, EXHN3, ST1, GB20, LI4, LI11, GV23 Certified practitioners with at least 7 years of traditional Korean medicine education and 3 years of clinical experience performed the acupuncture treatment.	Needle: 0.20*30 mm disposable acupuncture needles (Dongbang Co., Korea) Deep insertion: 0.6 to 3 cm for the acupuncture points at the face and head. 0.6 to 4.5 cm at the hand and arm. Stimulation: Manual acupoint stimulation until getting Qi.	20 min per session. Three times per week for four weeks.
Liu Q, et al. (2017) (60)	BL1, BL2, SJ23, EXHN5, ST2, LI4, GB20, GV20, ST1. No data concerning the practitioner’s background.	Needle: No data Deep insertion: No data	30 min per session. Three times per week for eight weeks.
Tong L, et al. (2018) (37)	ST1, BL2, GB20, EXHN5, SP6, LI4, ST36. No data concerning the practitioner’s background.	Needle: For eyes 0.25*13mm; for ear 0.25*25mm; for upper and lower limbs 0.30*25mm Deep insertion: 1–2 mm	20 min per session Two times per week for 30 days.
Dhaliwal, et al. (2019) (38)	Auricular acupuncture: Salivary Gland 2, Point Zero, Shen Men. Acupuncture corporal: LI1, LI2, and at a point between LI1 and LI2. No data concerning the practitioner’s background.	Needle: No. 3 gauge 0.20*30 mm (Seirin-America, Weymouth, MA, USA) Deep insertion: No data	45 min per session Two times per week A total of two sessions
Zhang X, et al. (2022) (27)	BL1 Acupuncturists with at least 5 years of acupuncture experience and trained before administering treatments in this study.	Needle: 0.3*40 mm (Huatuo) Deep insertion: No data	Three times per week for eight weeks.
Yang G, et al. (2022) (28)	BL2, SJ23, EXHN5, ST2, GV20, GB20, LI4, ST36, GB23, SP6, LR3. No data concerning the practitioner’s background.	Needle: Periocular acupoints; 0.30*25mm Other acupoints; 0.30*40mm Deep insertion: No data Electroacupuncture stimulation: 1.0 to 2.0 mA (adjusted to patients’ tolerance) at 2 Hz	30 min per session Three times per week for four weeks
Zhou X, et al. (2022) (39)	SJ5, KI6, CV24, CV23, EX-HN5, BL2, SJ23, ST6. Acupuncture by a qualified and registered clinical acupuncturist, trained to maintain consistent performance.	Needle: 40 × 0.25 mm Deep insertion: no data. Stimulation: manual twirling, lifting, and thrusting motions.	30 min per session 20 sessions in 8 weeks
Duan H, et al. (2024) (40)	BL1, BL2, ST1, LI20, SI1, SI3 Acupuncture by skilled Chinese medicine physician. With 20 years of experience in acupuncture manipulation.	Needle: 0.3 × 15 mm Deep insertion: 3–15 mm Stimulation: none.	30 min per session Three times per week for four weeks
Tang N, et al. (2024) (29)	BL2, EX-HN5, ST2, LI4, SJ23, BL1, GB20, DU20 Acupuncturist with more than 2 years of clinical experience	Needle: 0.25 × 25 mm Deep insertion: no data. Stimulation: none.	30 min per session Three times per week for four weeks
Diabetic retinopathy			
Chiu, et al. (2011) (30)	GB37 Acupuncture by a qualified Traditional Chinese Medicine practitioner. (no more data)	Needle: 0.18 mm × 25 mm Deep insertion: 1 to 1.5 inch Stimulation: no data	30 min per session One session per day for three days
Macular degeneration			
Xia Y, et al. (2013) (31)	BL1, GB1, ST1, EXHN7, LR3. No data concerning the practitioner’s background.	Needle: 0.25*40 mm Deep insertion: 1.5 to 2 cun Stimulation: Manual acupoint stimulation until getting Qi.	30 min per session Twice times a week for two months.
Glaucoma			
Her JS, et al. (2010) (45)	Auricular acupressure (kidney, liver, and eye) No data concerning the practitioner’s background.	Tapping stimulation was administered using a 1-mm alloy ball. Stimulation: manual	Twice per day, 3 min each point for four weeks
Liu F, et al. (2013) (43)	BL1, GB37, EXHN5, LR2, LR3 No data concerning the practitioner’s background	Needle: filiform needles of 0.30–0.35 mm in diameter. Deep insertion: 0.5–0.8 cun Stimulation: no data	20 min per session One session per day, ten days for one course of treatment, two courses in total
Takayama S, et al. (2011) (42)	BL2, EXHN5, ST2, ST36, SP6, KI3, LR3, GB20, BL18, BL23 Licensed acupuncturists with 5 years of experience	Needle: stainless steel needles of 0.16–0.20*40 mm Deep insertion: 20 mm Stimulation: none	15 min one session No more data
Law SK, et al. (2015) (32)	BL2, GB37, LR3, LI4 Acupuncture was performed by an experienced, licensed acupuncturist (no more data).	Needle: 1 inch 36-gauge needles and 1/2 inch 38 gauge needles Deep insertion: No data	20 min per session 12 sessions over 6–12 weeks
Yeh TY, et al. (2016) (46)	BL61, BL62 No data concerning the practitioner’s background.	Needle: No data Deep insertion: No data Stimulation: TENS (no more data)	20 min per session One session
Leszczynska A, et al. (2018) (44)	BL2, EXHN3, SJ23, GB1, GV6, GB37. Acupuncture treatment was performed by an experienced, licensed acupuncturist (no more data).	Needle: stainless-steel needles (Seirin B-type) Deep insertion: No data	No data
Chen SY, et al. (2020) (47)	BL1 and EXHN7 No data concerning the practitioner’s background.	Needle: No. 34 with a spherical needle head Deep insertion: No data	20 min per session Two sessions per weekly for 2 weeks

Regarding adverse events in dry eye syndrome treatment, six studies reported none, while four did not report whether adverse events were assessed or occurred. On the other hand, Kim TH, et al. reported three cases of hematoma in the acupuncture group (22). Zhang X, et al. reported five adverse events (4 bleeding and 1 pain) (27). Yang G, et al. reported three cases of hematomas (28). Tang N, et al. reported that two patients developed subcutaneous hematoma and one patient had pain (29).

During treatment for diabetic retinopathy, no adverse reactions occurred (30). On the other hand, during the treatment for macular degeneration, 10 of 22 cases in the acupuncture group experienced bleeding and hematoma in the orbital region (31). Regarding adverse events in glaucoma treatment, one study reported none, while five studies did not report whether adverse events were assessed or whether they occurred. On the other hand, Law SK, et al. reported that 2 patients were withdrawn from the study because of needle sensitivity, and 1 experienced IOP (32).

A summary of the characteristics of the interventions is shown in Table 2. Additionally, the characteristics and results of the included studies are presented in Tables 3–5.

**Table 3:** Characteristics and results of the included studies of dry eye syndrome in the systematic review.

ID	Population	Groups	Results
Neep J, et al. (1998) (33)	Patients with dry eye (*n* = 114)	G1: AT (*n* = 30) G2: PT (*n* = 22) G3: ALT (*n* = 32) G4: SALT (*n* = 30)	Results of Schirmer test (*p* < 0.01) Results of BUT (*p* < 0.001) Results of drop frequency (*p* < 0.001) No numerical data (data were categorized)
Grönlund MA, et al. (2004) (21)	Patients with keratoconjunctivitis sicca (KCS) (*n* = 25)	G1: AT (*n* = 12) G2: Sham (*n* = 13)	Tear breakup time (range) *Before sessions (Baseline)* G1: RE = 6.6 (3–10); LE = 6.9 (3–10); G2: RE = 6.9 (3–10); LE = 7.1 (2–10) *After sessions (8 months)* G1: RE = 7.3 (4–10); LE = 7.0 (4–10); G2: RE = 7.2 (4–10); LE = 7.1 (3–10) Schirmer 1 test (range) *Before sessions (Baseline)* G1: RE = 5.2 (0–22); LE = 6.2 (0.5–25); G2: RE = 6.2 (0–19); LE = 3.6 (0–10) *After sessions (8 months)* G1: RE = 6.2 (0–24); LE = 7.7 (1–25); G2: RE = 6.8 (1–30); LE = 5.1 (1–22) Rose-Bengal staining (range) *Before sessions (Baseline)* G1: RE = 3.1 (2–6); LE = 4.1 (2–6); G2: RE = 4.6 (4–6); LE = 4.2 (1–7) *After sessions (8 months)* G1: RE = 2.7 (0–6)*; LE = 2.9 (1–8)*, G2: RE = 3.2 (2–6)*; LE = 3.0 (0–8)*
Tseng KL, et al. (2006) (34)	Patients with dry eye (*n* = 43 eyes)	G1: AT+PT (*n* = 17) G2: ET + PT (*n* = 17) G3: PT (*n* = 9)	Schirmer 1 test *Before sessions (Baseline)* G1: RE = 2.00 ± 1.26; LE = 2.31 ± 1.11; G2: RE = 2.56 ± 1.41; LE = 3.33 ± 1.30; G3: RE = 2.56 ± 1.33; LE = 3.57 ± 0.54 *After sessions (8 weeks)* G1: RE = 7.72 ± 0.88; LE = 7.48 ± 0.83; G2: RE = 5.89 ± 0.95; LE = 6.35 ± 0.99; G3: RE = 1.99 ± 1.16; LE = 3.13 ± 1.18 Tear breakup time *Before sessions (Baseline)* G1: RE = 7.60 ± 3.48; LE = 5.00 ± 1.95; G2: RE = 6.44 ± 4.11; LE = 6.08 ± 4.25; G3: RE = 6.67 ± 3.00; LE = 6.26 ± 3.20 *After sessions (8 weeks)* G1: RE = 7.65 ± 0.98; LE = 5.13 ± 0.95; G2: RE = 6.79 ± 1.03; LE = 5.76 ± 1.10; G3: RE = 6.38 ± 1.27; LE = 6.16 ± 1.32
Shin MS, et al. (2010) (36)	Patients with dry eye (*n* = 42)	G1: AT (*n* = 21) G2: Sham (*n* = 21)	Ocular surface disease index (OSDI) *Before sessions (Baseline)* G1: 52.90 ± 16.76; G2: 57.95 ± 15.75 *After (4 weeks)* G1: −17.61 ± 15.61; G2: −17.20 ± 18.81 Visual analog scale (VAS) *Before sessions (Baseline)* G1: 60.86 ± 17.34; G2: 61.86 ± 15.53 *After (4 weeks)* G1: −16.81 ± 23.61; G2: −16.24 ± 19.55
Gong L, et al. (2010) (35)	Patients with xerophthalmia (*n* = 42)	G1: AT (*n* = 20) G2: PT (*n* = 22)	Schirmer 1 test (mm) *Before sessions (Baseline) Changes after 1 h* G1: 1.60 ± 2.23; G2: 2.00 ± 3.04 *After sessions (Changes after 3 weeks)* G1: 1.75 ± 2.23; G2: 0.09 ± 1.34 Tear breakup time *Before sessions (Baseline) Changes after 1 h* G1: 0.4 ± 0.99; G2: 1.55 ± 1.84 *After sessions (Changes after 3 weeks)* G1: 0.4 ± 0.99; G2: 0.05 ± 0.49
Kim TH, et al. (2012) (22)	Patients with dry eye (*n* = 150)	G1: AT (*n* = 75) G2: PT (*n* = 75)	Ocular surface disease index (OSDI) *Before sessions (Baseline)* G1: 50.05 ± 21.63; G2: 52.75 ± 18.79 *After sessions (12 weeks)* G1: 33.90 ± 21.42; G2: 41.99 ± 21.54 Visual analog scale (VAS) *Before sessions (Baseline)* G1: 66.67 ± 19.18; G2: 67.52 ± 17.31 *After sessions (12 weeks)* G1: 42.79 ± 26.88; G2: 52.81 ± 24.12 Tear breakup time *Before sessions (Baseline)* G1: 6.19 ± 2.18; G2: 6.01 ± 1.98 *After sessions (12 weeks)* G1: 6.68 ± 2.85; G2: 5.89 ± 1.99 Schirmer 1 test *Before sessions (Baseline)* G1: 4.49 ± 2.56; G2: 4.16 ± 2.66 *After sessions (12 weeks)* G1: 5.95 ± 5.02; G2: 5.28 ± 4.07
Liu Q, et al. (2017) (60)	Patients with dry eye (*n* = 28)	G1: AT+PT (*n* = 14) G2: PT (*n* = 14)	Ocular surface disease index (OSDI) *Before sessions (Baseline)* G1: 48.83 ± 17.68; G2: 51.41 ± 16.25 *After sessions (Post)* G1: 24.55 ± 17.43; G2: 39.08 ± 18.68 Tear breakup time *Before sessions (Baseline)* G1: 2.64 ± 1.49; G2: 1.93 ± 1.30 *After sessions (Post)* G1: 4.57 ± 2.71; G2: 4.75 ± 1.74 Schirmer 1 test *Before sessions (Baseline)* G1: 4.93 ± 3.39; G2: 4.86 ± 2.52 *After sessions (Post)* G1: 5.89 ± 4.51; G2: 5.43 ± 2.91
Tong L, et al. (2018) (37)	Patients with dry eye (*n* = 150)	G1: AT+PT (*n* = 50) G2: PT (*n* = 50) G3: PT + HT (*n* = 50)	Schirmer 1 test (mm) *Before sessions (Baseline)* G1: 10.9 ± 7.8; G2: 11.7 ± 8.6; G3: 13.1 ± 10.8 *After sessions (4 weeks)* G1: 11.4 ± 8.3; G2: 12.4 ± 9.0; G3: 14.5 ± 10.2 Non-invasive Tear breakup time NIBUT (s) *Before sessions (Baseline)* G1: 7.58 ± 5.44; G2: 9.36 ± 6.34; G3: 8.02 ± 6.13 *After sessions (4 weeks)* G1: 6.91 ± 5.08; G2: 9.79 ± 6.33; G3: 8.28 ± 5.63
Dhaliwal, et al. (2019) (38)	Patients with dry eye (*n* = 49)	G1: AT (*n* = 24) G2: Sham (*n* = 25)	Tear breakup time (range) *Before sessions (Baseline)* G1: 3 ± 3; G2: 3 ± 2 *After sessions (6 months)* G1: 3 ± 3; G2: 2 ± 2 Schirmer 1 test *Before sessions (Baseline)* G1: 10 ± 8; G2: 9 ± 7 *After sessions (6 months)* G1: 11 ± 9; G2: 10 ± 6 Ocular surface grading *Before sessions (Baseline)* G1: 8 ± 4; G2: 6 ± 3 *After sessions (6 months)* G1: 7 ± 4; G2: 6 ± 2
Zhang X, et al. (2022) (27)	Patients with dry eye (*n* = 120)	G1: AT (*n* = 60) G2: PT (*n* = 60)	Tear breakup time *Before sessions (Baseline)* G1: 4.31 (95%CI 3.38–5.25); G2: 4.40 (95%CI 3.56–5.32) *After sessions (Change, 32 weeks)* G1: 0.46 (95%CI -0.14 – 2.46); G2: 0.12 (95%CI -0.43 – 1.89) Schirmer 1 test *Before sessions (Baseline)* G1: 4.37 (95%CI 3.37–5.25); G2: 3.82 (95%CI 2.84–4.86) *After sessions (Change, 32 weeks)* G1: 2.05 (95%CI 0.94–4.47); G2: 0.16 (95%CI -1.94 – 2.83) Ocular surface grading *Before sessions (Baseline)* G1: 4.31 (95%CI 3.38–5.25); G2: 4.40 (95%CI 3.56–5.32) *After sessions (Change, 32 weeks)* G1: −18.18 (95%CI -26.46 – 16.54); G2: −6.03 (95%CI -21.89 – 19.32)
Yang G, et al. (2022) (28)	Patients with dry eye (*n* = 84)	G1: ET (*n* = 42) G2: PT (*n* = 42)	Non-invasive Tear breakup time *Before sessions (Baseline)* G1: 4.72 ± 2.95; G2: 4.21 ± 1.68 *After sessions (8 weeks)* G1: 1.40 ± 0.31; G2: 0.15 ± 0.32 Schirmer 1 test *Before sessions (Baseline)* G1: 8.59 ± 6.22; G2: 8.47 ± 5.27 *After sessions (8 weeks)* G1: 2.12 ± 1.01; G2: 0.69 ± 1.03 Ocular surface grading *Before sessions (Baseline)* G1: 36.33 ± 21.00; G2: 36.57 ± 19.80 *After sessions (8 weeks)* G1: −19.02 ± 2.88; G2: −9.21 ± 2.92
Zhou X, et al. (2022) (39)	Patients with Primary Sjógren’s syndrome (*n* = 120)	G1: PT + AT (*n* = 60) G2 PT + Sham (*n* = 60)	Schirmer 1 test *Before sessions (Baseline)* G1: 2.88 ± 2.66; G2: 3.42 ± 2.71 *After sessions (8 weeks)* G1: 2.74 ± 2.12; G2: 3.04 ± 2.89
Duan H, et al. (2024) (40)	Patients with dry eye (*n* = 48)	G1: AT (*n* = 27) G2: Sham (*n* = 21)	Tear breakup time *Before sessions (Baseline)* G1: 3.85 ± 2.33; G2: 4.38 ± 2.48 *After sessions (4 weeks)* G1: 5.63 ± 3.58; G2: 5.10 ± 3:00 Ocular surface disease index (OSDI) *Before sessions (Baseline)* G1: 49.75 ± 14.92; G2: 44.39 ± 20.13 *After sessions (4 weeks)* G1: 29.64 ± 18.79; G2: 25.82 ± 18.09
Tang N, et al. (2024) (29)	Patients with video display terminal related dry eye (*n* = 60)	G1: AT (*n* = 30) G2: PT (*n* = 30)	Ocular surface disease index (OSDI) *Before sessions (Baseline)* G1: 36.29 ± 17.61; G2: 30.16 ± 15.52 *After sessions (4 weeks)* G1: 22.38 ± 11.41; G2: 26.96 ± 14.56 Non-invasive Tear breakup time *Before sessions (Baseline)* G1: 3.46 ± 1.46; G2: 4.03 ± 1.59 *After sessions (4 weeks)* G1: 4.73 ± 2.56; G2: 4.56 ± 2.09 Schirmer 1 test *Before sessions (Baseline)* G1: 5.77 ± 4.08; G2: 7.96 ± 7.88 *After sessions (4 weeks)* G1: 10.70 ± 8.73; G2: 11.90 ± 9.84

AT, Acupuncture; ET, Electroacupuncture; WT, Without treatment; PT, Pharmacological treatment; ALT, Acupuncture with laser; SALT, Sham acupuncture with laser; HT, Herbal treatment; RE, Right eye; LE, Left eye; OSDI, Ocular surface disease index; TFBUT, Tear film breakup time; VAS, Visual analog scale; QOL, Quality of life.

**Table 4:** Characteristics and results of the included studies of macular degeneration in the systematic review.

ID	Population	Groups	Results
Xia Y, et al. (2013) (31)	Patients with age-related macular degeneration (*n* = 47)	G1: AT (*n* = 22) G2: PT (*n* = 15) G3: WT (*n* = 10)	Age-related macular degeneration; eye symptoms before and after treatment (Mean, SD). Blurred vision (eyes) *Before treatment* G1: 3.00 ± 0.00 (*n* = 44); G2: 3.00 ± 0.00 (*n* = 30); G3: 3.00 ± 0.00 (*n* = 20) *After treatment (3 months)* G1: 2.00 ± 0.03 (*n* = 44); G2: 2.90 ± 0.04 (*n* = 30); G3: 3.00 ± 0.00 (*n* = 20) Drastic decrease in visual acuity (eyes) *Before treatment* G1: 3.00 ± 0.00 (*n* = 32); G2: 3.00 ± 0.00 (*n* = 25); G3: 3.00 ± 0.00 (*n* = 16) *After treatment (3 months)* G1: 1.00 ± 0.01 (*n* = 32); G2: 3.00 ± 0.00 (*n* = 25); G3: 3.00 ± 0.00 (*n* = 16) Distorted vision (eyes) *Before treatment* G1: 3.00 ± 0.00 (*n* = 10); G2: 3.00 ± 0.00 (*n* = 8); G3: 3.00 ± 0.00 (n = 6) *After treatment (3 months)* G1: 1.00 ± 0.02 (n = 10); G2:3.00 ± 0.00 (n = 8); G3: 3.00 ± 0.00 (*n* = 6) Central scotomas (eyes) *Before treatment* G1: 3.00 ± 0.00 (*n* = 32); G2: 3.00 ± 0.00 (*n* = 20); G3: 3.00 ± 0.00 (*n* = 14) *After treatment (3 months)* G1: 1.00 ± 0.01 (*n* = 32); G2: 3.00 ± 0.00 (*n* = 20); G3: 3.00 ± 0.00 (n = 14) Visual fatigue (eyes) *Before treatment* G1: 3.00 ± 0.00 (*n* = 36); G2: 3.00 ± 0.00 (*n* = 30); G3: 3.00 ± 0.00 (*n* = 18) *After treatment (3 months)* G1: 1.00 ± 0.02 (*n* = 36); G2: 3.00 ± 0.00 (*n* = 30); G3: 3.00 ± 0.00 (*n* = 18) Seeing things in changed colors (eyes) *Before treatment* G1: 3.00 ± 0.00 (*n* = 8); G2: 3.00 ± 0.00 (*n* = 6); G3: 3.00 ± 0.00 (*n* = 4) *After treatment (3 months)* G1: 2.50 ± 0.03 (*n* = 8); G2: 3.00 ± 0.00 (*n* = 6); G3: 3.00 ± 0.00 (*n* = 4) Dry eyes (eyes) *Before treatment* G1: 3.00 ± 0.00 (*n* = 24); G2: 3.00 ± 0.00 (*n* = 18); G3: 3.00 ± 0.00 (*n* = 12) *After treatment (3 months)* G1: 1.00 ± 0.01 (*n* = 24); G2: 3.00 ± 0.00 (*n* = 18); G3: 3.00 ± 0.00 (*n* = 12)

AT, Acupuncture; ET, Electroacupuncture; WT, Without treatment; PT, Pharmacological treatment.

**Table 5:** Characteristics and results of the included studies of glaucoma in the systematic review.

ID	Population	Groups	Results
Her JS, et al. (2010) (45)	Patients with glaucoma (*n* = 33)	G1: AT (n = 16) G2: Sham (*n* = 17)	Intraocular pressure (mmHg) IOP *After (Baseline)* G1: RE = 16.7 ± 3.9; LE = 17.3 ± 4.2; G2: RE = 18.4 ± 7.0; LE = 19.7 ± 6.8 *Before (8 weeks)* G1: RE = 15.8 ± 4.2; LE = 16.3 ± 4.3; G2: RE = 17.1 ± 5.9; LE = 18.6 ± 5.7 Visual acuity (logMAR) UCVA *After (Baseline)* G1: RE = 0.53 ± 0.49; LE = 0.49 ± 0.41; G2: RE = 0.48 ± 0.36; LE = 0.64 ± 0.54 *Before (8 weeks)* G1: RE = 0.40 ± 0.37; LE = 0.35 ± 0.37; G2: RE = 0.43 ± 0.31; LE = 0.51 ± 0.39
Liu F, et al. (2013) (43)	Patients with Primary Open Angle Glaucoma (*n* = 19)	G1: AT+PT (*n* = 10) G2: PT (*n* = 9)	Intraocular pressure (mmHg) *Before treatment* G1: 24.45 ± 4.95; G2: 25.12 ± 4.72 *After treatment (3 months)* G1: 21.06 ± 2.76; G2: 23.03 ± 3.53
Takayama S, et al. (2011) (42)	Patients with open-angle glaucoma (*n* = 11)	G1: PT + AT (*n* = 11) G2: PT (*n* = 11) (same patients)	Intraocular pressure (mmHg) *Before treatment* G1: 17.0 ± 5.0; G2: 16.0 ± 4.1 *After treatment (4 weeks)* G1: 16.0 ± 4.3; G2: 17.1 ± 4.2 Retrobulbar circulation (Ophthalmic artery) *Before treatment* G1: 0.74 ± 0.04; G2: 0.74 ± 0.04 *After treatment (4 weeks)* G1: 0.74 ± 0.04; G2: 0.75 ± 0.05 (Central retinal artery) *Before treatment* G1: 0.72 ± 0.05; G2: 0.75 ± 0.09 *After treatment (4 weeks)* G1: 0.68 ± 0.04; G2: 0.72 ± 0.03 (Short posterior ciliary artery) *Before treatment* G1: 0.67 ± 0.04; G2: 0.68 ± 0.05 *After treatment (4 weeks)* G1: 0.64 ± 0.06; G2: 0.68 ± 0.04
Law SK, et al. (2015) (32)	One eye per patient with primary open-angle glaucoma and stable intraocular pressure (*n* = 11)	G1: AT+PT (*n* = 11) G2: Sham+PT (*n* = 11) (only one eye) G1 = G2	Intraocular pressure (mmHg) *Before treatment* G1: 12.9 ± 1.8; G2: 13.0 ± 1.5 *After individual treatment session (6–12 weeks)* G1: 13.6 ± 2.1; G2: 13.5 ± 1.7 Best-corrected visual acuity (logMAR) *Before treatment* G1: 0.084 ± 0.138; G2: 0.102 ± 0.125 *After 12 treatments* G1: 0.084 ± 0.138; G2: 0.097 ± 0.140 Diurnal intraocular pressure (mmHg) *Before treatment* G1: 12.90 ± 2.0; G2: 13.1 ± 1.8 *After 12 treatments* G1: 12.7 ± 2.0; G2: 12.8 ± 1.8
Yeh TY, et al. (2016) (46)	Patients with glaucoma (*n* = 82)	G1: ET (*n* = 40) G2: Sham (*n* = 42)	Intraocular pressure (mmHg) Effect after stimulation (60 min) G1: −3.45 ± 1.51; G2: 0.57 ± 1.84
Leszczynska A, et al. (2018) (44)	Patients with primary open-angle glaucoma (*n* = 56)	G1: AT (*n* = 28) G2: Sham (*n* = 28)	Intraocular pressure (mmHg) *Before treatment* G1: 16.1 ± 4.5; G2: 16.4 ± 4.5 *After treatment (10 min)* G1: 15.3 ± 5.7; G2: 16.5 ± 4.8 Pulse amplitude (mmHg) *Before treatment* G1: 3.4 ± 1.0; G2: 4.3 ± 1.9 *After acupuncture* G1: 3.7 ± 1.5; G2: 4.4 ± 2.1 Pulsatile OBF (μL/min) *Before treatment* G1: 5.6 ± 4.3; G2: 6.3 ± 3.6 *After treatment* G1: 6.7 ± 4.9; G2: 6.8 ± 4.7
Chen SY, et al. (2020) (47)	Patients with glaucoma (*n* = 45)	G1: AT (*n* = 15) G2: ET (*n* = 15) G3: Sham (*n* = 15)	Intraocular pressure (mmHg) *Before treatment* G1: RE = 18.37 ± 3.91; G2: RE = 19.77 ± 3.44; G3: RE = 16.95 ± 5.34 G1: LE = 19.34 ± 3.91; G2: LE = 20.66 ± 3.44; G3: LE = 16.88 ± 3.98 *After treatment (2 weeks)* G1: RE = 15.06 ± 3.78; G2: RE = 15.22 ± 3.02; G3: RE = 17.17 ± 4.86 G1: LE = 15.19 ± 3.75; G2: LE = 15.37 ± 3.05; G3: LE = 16.86 ± 3.32

AT, Acupuncture; ET, Electroacupuncture; WT, Without treatment; PT, Pharmacological treatment; ALT, Acupuncture with laser; SALT, Sham acupuncture with laser; HT, Herbal treatment; RE, Right eye; LE, Left eye; VAS, Visual analog scale; QOL, Quality of life; MYMOP-2, Measure Yourself Medical Outcome Profile-2; IOP, Intraocular pressure.

### 3.2 Dry eye syndrome

**Figure 3:**
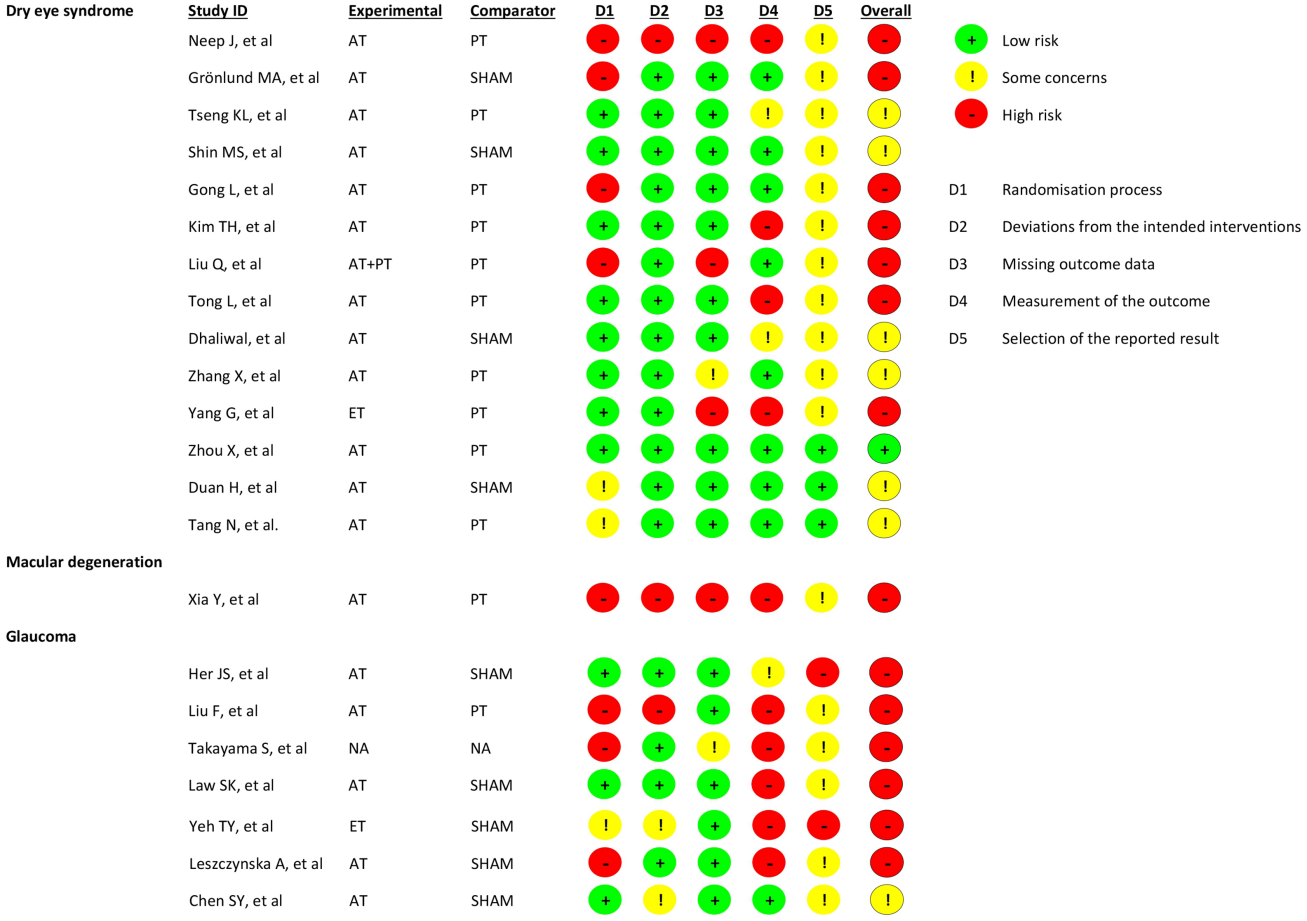
Risk of bias.

Fourteen studies met the eligibility criteria. The main outcomes assessed were the Schirmer 1 test and the tear breakup time. In terms of the overall risk of bias of the included studies, 50.0% exhibited a high risk, 42.8% had some concerns, and 7.1% showed low risk of bias. The main problems were identified in the following domains: the randomization process and the measurement of outcomes, as shown in Figure 3.

#### 3.2.1 Population characteristics

Neep J, et al. included Patients with S1T below 5 mm and BUT below 5 s (33); Grönlund MA, et al. included patients diagnosed via Copenhagen criteria (primary, secondary and not associated Sjögren’s syndrome were included) (21), Tseng KL, et al. do not differentiate between DES diagnoses (34); Gong L, et al. included patients with xerophthalmia (35), Shin MS, et al. included patients with moderate dry eye symptoms (BUT below 10 s and with a S1T with anesthesia below 10 mm), also (36), Kim TH, et al. included patients with de same criteria. Liu, et al. focused in postmenopausal women diagnosed with DES (22); Tong L, et al. used Traditional Chinese Medicine (TCM) diagnosis Lung–kidney yin deficiency (37); Dhaliwal, et al. included patients with “previous diagnosis of dry eye disease by an ophthalmologist” without more description (38); Zhang X, et al. included patients with moderate to severe DES (Symptom severity ≥4/10 on Numerical Rating Scale) (27); Yang G, et al. included patients with objective DED symptoms plus objective signs (SIT ≤5 mm/5 min or non-invasive TBUT ≤5 s; or 5 < SIT ≤10 mm/5 min or 5 < NIBUT ≤10 s + positive CFS) (28); Zhou X, et al. included older, exclusively systemic autoimmune patients with primary Sjögren’s syndrome (39); Duan H, et al. included patients with DED diagnosed according to the Tear Film and Ocular Surface Society DEWS II criteria and numerical rating scale score >1 (40); finally Tang N, et al. focused in patients with video display terminal-related dry Eye (29). The diagnostic criteria for DES varied significantly across studies, reflecting the heterogeneous patient populations and methodological approaches employed. This heterogeneity highlights challenges in synthesizing evidence for DES interventions, particularly for acupuncture, where population characteristics have a significant influence on outcomes. Nevertheless, Given the predominantly high risk of bias observed across the majority of included studies, a quantitative synthesis via meta-analysis was deemed methodologically inappropriate. Critical methodological flaws, particularly in outcome assessment and the selection of reported results, fundamentally compromised the validity and comparability of effect estimates, precluding meaningful statistical pooling. Consequently, a narrative synthesis was employed exclusively to contextualize the findings.

Four studies reported acupuncture as having significant improvements compared to controls. Neep J, et al. evaluated acupuncture for dry eye treatment across four groups: verum laser acupuncture, sham laser acupuncture, needle acupuncture, and artificial tears-only controls. After 10 weekly sessions, objective improvements were observed in the verum laser and needle acupuncture groups compared to controls. Specifically, both active interventions significantly increased Schirmer test scores (*p* < 0.01) and tear film breakup time (*p* < 0.001). The verum laser group outperformed the sham laser group (*p* < 0.01), confirming physiological efficacy beyond placebo. Similarly, needle acupuncture significantly surpassed the control group. Crucially, no significant difference existed between verum laser and needle acupuncture, suggesting comparable therapeutic effects. The sham laser and control groups showed no statistically significant difference, underscoring the specificity of the active interventions (33). Tseng KL, et al. studied DES patients divided into three groups: a control group receiving only artificial tears, an acupuncture group, and an electroacupuncture group. After 8 weeks, both intervention groups showed significantly greater improvements than the controls in Schirmer test scores(*p* < 0.01) and Visual Analog Scale scores (*p* < 0.01), reflecting symptomatic relief. However, no significant changes occurred in tear breakup time for any group (34). Gong L, et al. included two study groups: an acupuncture therapy group and a control group using artificial tears. Assessments using Symptom and Sign Scores, S1T, and BUT suggested Immediate effects (1 h post-treatment) in both groups, showing reduced symptoms. However, BUT improved more in the artificial tears group (0.4 ± 0.99 vs. 1.55 ± 1.84; *p* = 0.0180). Also, the results suggested a sustained effect (3 weeks post-treatment) (35); Duan H, et al. divided patients into two intervention groups: an acupuncture group and a control group using eye drops. Key directional outcomes revealed that both groups showed significant reductions in ocular pain (as measured by the NRS score, both *p* < 0.05 compared with baseline) and dry eye symptoms (as measured by the OSDI score). However, only acupuncture demonstrated significant improvements in the TBUT (*p* < 0.05), corneal sensitivity (*p* < 0.05), mental health (reduced anxiety/depression scores SAS/SDS) (*p* < 0.05), and inflammatory cytokines such as IL-1β, IL-6 and TNF-*α* (*p* < 0.05) (40).

Conversely, other studies reported inconsistencies in the direction of effect. Grönlund MA, et al. evaluated acupuncture in DES patients, allocating them to either an acupuncture group receiving 10 standardized sessions plus artificial tears, or a control group receiving artificial tears alone. At the first follow-up (2–3 weeks post-treatment), subjective improvement (VAS) was significantly greater in the acupuncture group (*p* = 0.036). Though this effect was not sustained at the 8-month follow-up. Objective measures, including BUT, S1T, and Rose Bengal staining (RBS), revealed no significant differences between the groups (21). Shin MS, et al. evaluated patients assigned to either the acupuncture or sham acupuncture groups. While within-group analyses revealed statistically significant improvements from baseline in subjective symptoms (OSDI and VAS scores) for both groups (*p* < 0.01), and in BUT for the acupuncture group (*p* = 0.0044), no statistically significant differences were found between the acupuncture and sham groups for any of the primary outcomes (OSDI, VAS (*p* = 0.84), BUT (*p* = 0.25), or Schirmer I test (*p* = 0.54)). Both interventions demonstrated clinically meaningful improvements in OSDI, exceeding the minimal clinically important difference; however, the magnitude of change did not differ significantly between groups. The findings suggest that while acupuncture treatment was associated with symptomatic relief and improved BUT, the specific needling protocol used did not yield superior outcomes compared to the sham procedure (36). Kim TH, et al. assigned patients to either acupuncture or artificial tears groups. While both groups showed clinically meaningful improvements in subjective symptoms (OSDI and VAS) during the 4-week treatment period with no significant between-group differences (*p* = 0.41), the acupuncture group demonstrated significantly greater mid-term benefits at 8-week follow-up for both OSDI (*p* = 0.030) and VAS (*p* = 0.018). Acupuncture also yielded superior objective outcomes in tear film stability at the end of treatment, and sustained improvement in the right eye at follow-up. However, no significant differences were observed in the Schirmer test or quality of life measures at any time point (22). Tong L, et al. allocated patients into three parallel groups, artificial tears; acupuncture plus artificial tears, and herbal formulation plus artificial tears. The research team reported 88% of acupuncture participants showed symptom improvement compared to 72% in the artificial tears group. However, no significant differences were observed in tear osmolarity, evaporation rates, Schirmer I, corneal staining, or non-invasive tear breakup time between groups (37). Dhaliwal, et al. assigned participants with dry eye disease to either true acupuncture or sham at non-meridian points. Both groups received two consecutive daily sessions. Subjective outcomes (OSDI scores) improved significantly in the true acupuncture group at all follow-ups (1 week to 6 months; *p* < 0.05), with a notable between-group difference favoring true acupuncture at 6 months. True acupuncture also significantly reduced symptoms of dryness, scratchiness, and redness in intragroup analysis and decreased the frequency of eye closing. However, objective measures (tear breakup time, Schirmer’s test, ocular surface staining) showed no significant improvement in either group. The results indicate that true acupuncture provides sustained subjective symptom relief beyond placebo effects, although without altering objective clinical signs of dry eye (38). Zhang X, et al. assigned patients to either acupuncture or artificial tears for 8 weeks, followed by a 24-week follow-up. For the primary outcome, acupuncture significantly increased tear secretion compared to artificial tears at weeks 4 (*p* < 0.001) and 8 (*p* = 0.01), but not at week 32. Acupuncture also demonstrated greater improvement in symptom burden (OSDI score) versus artificial tears at week 8 (*p* = 0.036). However, no significant between-group differences were observed in average symptom intensity, tear breakup time, or corneal staining. Acupuncture was well-tolerated (8.3% mild–moderate adverse events) (27); Yang G, et al. allocated patients to either an electroacupuncture group or an artificial tears group. Results showed significantly greater improvements in tear film stability (*p* = 0.008), tear volume (*p* = 0.009), OSDI (*p* = 0.022), and corneal damage (*p* = 0.036) with electroacupuncture compared to artificial tears at week 8. No significant between-group differences were observed in Schirmer I test, corneal topography indices (SAI/SRI), corneal sensitivity, or mood scores (HADS) (28); Zhou X, et al. enrolled primary Sjögren’s syndrome patients, assigning them to either true acupuncture or sham acupuncture. No significant difference was found in the primary outcomes. Secondary outcomes, including EULAR symptom scores (ESSPRI), quality of life (SF-36), anxiety and depression (HADS), tear production (Schirmer test), and salivary flow, also showed no between-group differences. Both groups exhibited similar trends toward symptom reduction, suggesting no superiority of true acupuncture over sham for core symptoms (39); Tang N, et al. divided patients with video display terminal-related dry eye into two groups: an acupuncture group and an artificial tear group. The research group reported that both groups showed reduced dry eye symptoms (OSDI scores), but only acupuncture demonstrated a significantly greater reduction in OSDI scores compared to artificial tears at 4 weeks post-treatment (*p* = 0.017). Tear film breakup time (TFBUT) and Schirmer I test (SIT) improved in the acupuncture treatment group, but the differences did not reach statistical significance between groups as shows in Table 3 (29).

Finally, the overall risk of bias across studies was high, leading to a very serious risk of bias. According to GRADE criteria, this limitation downgraded the certainty of evidence by two levels (from high to low). Additionally, the inconsistency in the direction of the effect across studies downgrades the certainty of evidence by one level (from low to very low), as shown in Table 6.

**Table 6:** Quality assessment of the included studies.

Certainty assessment								
No of studies	Study design	Risk of bias	Inconsistency	Indirectness	Imprecision	Other considerations	Certainty	Importance
Dry eye syndrome								
14	Clinical trials	very serious	serious	not serious	not serious	none	⨁◯◯◯ Very low	Important
Macular degeneration								
1	Clinical trial	very serious	—	not serious	not serious	none	⨁⨁◯◯ Low	Critical
Glaucoma								
7	Clinical trial	very serious	not serious	not serious	not serious	none	⨁⨁◯◯ Low	Important

### 3.3 Diabetic retinopathy

The search yielded 22 records concerning DR treated with acupuncture. Two of these were related to the research question (30, 41); however, none fully met the eligibility criteria.

### 3.4 Macular degeneration

The search yielded seven records concerning maculopathy treated with acupuncture. However, only one met the eligibility criteria. Xia Y, et al. (31) performed an RCT with three groups of patients (acupuncture, western medicine, and without treatment groups) with macular degeneration. The group treated with Western medicine receives Vitamin C and E, three daily doses for two months. The eye symptoms (blurred vision, drastic decrease in visual acuity, distorted vision, central scotomas, visual fatigue, seeing things in changed colors, and dry eyes) were scored. At the end of the follow-up, the research team reported that eye symptoms were significantly improved in the acupuncture group (*p* < 0.05), as shown in Table 4. However, this study showed a high risk of bias, as shown in Figure 3.

### 3.5 Glaucoma

The search yielded 21 records related to the research question; only 7 met the eligibility criteria. In terms of the overall risk of bias of the included studies, 85.7% exhibited high risk, and 14.2% had some concerns. The main problems were in the following domains: randomization process and the measurement of the outcomes, as shown in Figure 3.

Four studies included patients with primary OAG (32, 42–44). However, Her JS, et al. included patients diagnosed with primary open-angle glaucoma (POAG), patients diagnosed with chronic angle-closure glaucoma (CACG), and patients diagnosed with intraocular hypertension (OHT) (Elevated intraocular pressure without any detectable signs of glaucomatous optic nerve damage) (45). Ye TY, et al. included patients with glaucoma and those with OHT (46). Finally, Chen SY, et al. did not differentiate between primary and secondary or open-angle and closed-angle glaucoma diagnoses (47). These patient characteristics introduce high heterogeneity among the studies.

#### 3.5.1 Primary glaucoma

Takayama S, et al. conducted a study involving 11 patients with OAG (20 eyes) who were stabilized on topical medications for at least 3 months. Participants received a single bilateral acupuncture session at specific points. Retrobulbar hemodynamics (using color Doppler imaging) and IOP were measured before and after acupuncture and were compared to control measurements taken at rest and 1 h post-rest. Results demonstrated a significant reduction in the resistive index (vascular resistance) in the short posterior ciliary artery (*p* < 0.01) and a significant decrease in IOP post-acupuncture compared to control (*p* < 0.05). While central retinal artery resistance also decreased after acupuncture, systemic hemodynamics (blood pressure and heart rate) remained unchanged. These findings indicate that acupuncture acutely improves choroidal perfusion and lowers IOP in medicated OAG patients, suggesting its potential as an adjunctive therapy. However, limitations of this study include its single-session design and small sample size (42).

Liu F, et al. divided participants into a treatment group receiving combined therapy (acupuncture plus eye drops twice daily) and a control group receiving eye drops alone. IOP was measured at baseline, 1 month, and 3 months. Results showed significant IOP reductions in both groups at 1 and 3 months compared to baseline (*p* < 0.05). However, the treatment group exhibited significantly greater IOP reduction than the control group at both time points (*p* < 0.05), indicating that combining acupuncture with topical medication is more effective for IOP control in POAG than eye drops alone (43).

Conversely, two studies reported no significant changes in IOP. Law SK, et al. allocated participants in two groups, acupuncture or sham acupuncture. Results revealed a small but statistically significant transient IOP increase immediately after individual eye-point sessions (*p* = 0.019). However, no sustained IOP changes were observed after completing either 12-session series (*p* = 0.711). No significant changes occurred in best-corrected visual acuity, visual field indices, optic disc parameters, or retinal nerve fiber layer thickness. Adverse events included needle sensitivity (2 patients) and IOP elevation in the contralateral eye (1 patient), with a 50% dropout rate (11/22 enrolled). The study concluded that acupuncture lacks sustained IOP-lowering effects in glaucoma (32).

Leszczynska A, et al. assessed patients to an acupuncture group or an eye-unspecific acupuncture group as sham acupuncture. Ocular blood flow (OBF) parameters were measured before and 10 min after a single acupuncture session (parapapillary retinal flow, retinal vessel diameter, and IOP). Results showed a significant increase in pulsatile OBF in the acupuncture group (*p* = 0.014), indicating improved choroidal perfusion. No significant changes occurred in retinal blood flow (*p* = 0.113), retinal vessel diameter (*p* = 0.220), ou IOP (*p* = 0.881) in either group as shown in Table 5 (44).

#### 3.5.2 General glaucoma

Her JS, et al. divided glaucoma patients into an auricular acupressure group and a sham group. In their results, the acupressure group showed statistically significant IOP lowering compared to baseline at 10 min (*p* < 0.05) and weeks 1–4 post-treatment. (*p* < 0.01) IOP returned to baseline 4 weeks after discontinuation (week 8). The sham group showed no significant changes in IOP. Also, Uncorrected VA (UCVA) significantly improved in the acupressure group at weeks 2–4 (right eye) and weeks 3–4 (left eye) (*p* < 0.05). Best-corrected VA (BCVA) improved significantly only at week 2 (right eye). The sham group showed limited, non-significant UCVA/BCVA improvements except transient UCVA gain (left eye, week 3). Effects were not sustained after discontinuation, suggesting that ongoing treatment is needed (45).

Yeh TY, et al. allocated participants to an experimental group (*n* = 40) receiving transcutaneous electrical nerve stimulation (TENS) with direct current (DC) at acupoints, or into a control group receiving sham TENS (electrodes without current). IOP was measured before, immediately after, and at 30/60 min post-intervention. Results showed significant IOP reductions in the experimental group compared to the control at all time points (*p* < 0.001) (46). Chen SY, et al. divided patients into three groups: a sham group, an acupuncture group, and an electroacupuncture group. The results showed that both acupuncture and electroacupuncture demonstrated significantly greater reductions in IOP compared to the sham group at 60 min post-intervention across all four treatment sessions and in both eyes (*p* < 0.001) (47).

The overall risk of bias across studies was high, resulting in a very serious risk of bias. According to GRADE criteria, this limitation downgraded the certainty of evidence by two levels (from high to low), as shown in Table 4.

## 4 Discussion

Dry eye syndrome is a condition that results from various causes, such as reduced tear production, increased evaporation, or poor-quality tears. Chronic instability of tears can lead to inflammation, ocular surface damage, and abnormal pain perception. Also, elevated levels of pro-inflammatory cytokines such as IL-6 and TNF-*α* and oxidative stress markers such as malondialdehyde (MDA) are often found in the tears of dry eye patients; in consequence, anti-inflammatory treatments such as cyclosporine, lifitegrast, and antioxidants are being explored to break this cycle and improve dry eye symptoms (4). Nevertheless, acupuncture has been reported to alleviate oxidative stress and inflammation in patients, downregulating MDA, serum nitrate and nitrite, serum C-reactive protein, IL-6, IL-17, TNF-α levels, and glutathione peroxidase (40, 48, 49). Wei, et al. performed a systematic review of RCT of acupuncture for treating dry eye syndrome, including 10 Chinese articles. The review team’s results suggested that acupuncture in periocular acupoints plus body acupoints improves the S1T (WMD = 1.98, 95% CI: 0.44 to −3.34, *p* < 0.00001), BUT (WMD = 1.01, 95% CI: 0.56 to −1.84, *p* < 0.00001), and symptoms score (WMD = -1.02, 95% CI: −1.33 to −0.72, *p* < 0.00001) in patients with dry eye syndrome; however, the risk of bias of the included studies using ROB 2 ranged from “some concerns” to “high” (50), and all studies were included in the meta-analysis even with a high risk of bias, which is not appropriated.

DR is a microvascular disease involving an abnormality in the retinal blood vessels, leading to visual impairment or complete vision loss. The early stages of the disease are characterized by retinal microaneurysms, microhemorrhages, cotton wool spots, and exudates. ROS damage the retinal vascular endothelium, leading to increased permeability and breakdown of the blood-retinal barrier. Later, it shows fragile new blood vessels and fibrotic membranes in the chronic stages, contributing to blinding eye disease (51). Most registers concerning DR and acupuncture in this review were related to the pain management associated with the panretinal photocoagulation. None of the articles were related to the present review’s focused question or met the eligibility criteria. Ang L, et al. performed a systematic review of RCT of acupuncture for treating retinopathy, including six Chinese articles. The review team’s results suggested that acupuncture combined with standard therapy improves the effective rate and visual acuity in patients with DR; however, the quality of the included studies ranged from “low” to “moderate” according to the GRADE evaluation system (52), which raises doubts about the magnitude of the treatment effect, since an overestimation of the effect usually occurs when low-quality primary studies are used.

AMD is an inflammatory disease that can be classified based on clinical characteristics into dry (non-vascular) AMD and wet AMD (neovascular) characterized by neovascularization in the macula (53). Oxidative stress contributes to the degeneration of retinal cells and structures, particularly the retinal pigment epithelium (RPE) and photoreceptors. The RPE supports photoreceptor function by phagocytosing shed photoreceptor outer segments and recycling visual pigments. Oxidative stress impairs RPE function, accumulating lipofuscin, a byproduct of incomplete digestion of photoreceptor outer segments that generate ROS when exposed to light, further damaging the RPE (54). In this review, only one article that suggests acupuncture improves AMD symptoms met the eligibility criteria; however, this study showed a high risk of bias. Sun W, et al. performed a meta-analysis that included nine articles in Chinese language with a total of 508 patients with AMD. Their results of clinical response rates (RR = 1.29, 95% CI: 1.17 to 1.42), best-corrected visual acuity (SMD = 0.95, 95% CI: 0.26 to 1.64), central macular thickness reduction (MD = −32.74, 95% CI: −60.96 to −4.55) suggested that acupuncture is a good treatment for AMD patients; however, the quality of the included studies ranged from “low” to “very low” according to the GRADE system (55).

Glaucoma is a chronic, progressive ocular degenerative disease associated with optic neuropathies and alteration of retinal ganglion cells (RGC). ROS damages cellular components, including lipids, proteins, and DNA, leading to RGC apoptosis related to increased intraocular pressure (56). Law SK et al., in a systematic review, reported that the between-group difference in intraocular pressure was −3.70 (95% CI: −7.11 to −0.29) for the right eye and −4.90 (95% CI: −8.08 to −1.72) for the left eye in patients treated with acupuncture versus control. However, the studies included showed very low quality, according to GRADE (57).

Most of the systematic reviews related to managing dry eye syndrome, diabetic retinopathy, AMD, and glaucoma with acupuncture are systematic reviews that include full text in Chinese, which may cause a language bias. Unfortunately, some Chinese-language trials do not provide helpful data due to methodological flaws (58). Nevertheless, data from trials with a high risk of bias provide a low or very low quality of evidence that prevents their use in a meta-analysis since their inclusion will generate inaccurate results. Therefore, improvements in acupuncture trials are needed to develop robust evidence that achieves international quality standards (59).

## 5 Limitations

The absence of eligible studies for DR, the inclusion of only one AMD study, and the limited availability of high-quality evidence for others underscore a critical gap in the current literature. This highlights the urgent need for well-designed, methodologically rigorous primary studies investigating the effects of acupuncture in the context of inflammation-related ocular degenerative diseases.

A key limitation of this review is that a meta-analysis could not be performed justifiably due to the pervasive risk of bias identified in more than 90% of the included studies. Concerns introduced substantial heterogeneity, threatening the validity of any pooled effect estimates. Consequently, findings are presented as a narrative synthesis to avoid misleading quantitative conclusions.

The absence of studies meeting the inclusion criteria for diabetic retinopathy, coupled with the inclusion of only a single low-quality trial for macular degeneration, severely compromises the validity of our analysis for these conditions. Consequently, no reliable evidence can be derived, and clinical inferences are entirely unwarranted.

Low incidence of adverse effects, limited to minor events such as hematomas, was reported; however, 41% of the included studies did not report whether adverse events were assessed or whether they occurred. Also, 60% of the included studies did not report the qualifications of acupuncture practitioners, which makes it impossible to analyze whether the direction of the effect on results or adverse events depends on the acupuncturist’s experience.

Even with language restrictions, most studies have been conducted in Asia (73%), which limits geographical representation and the predominance of studies in Chinese populations. This limited geographical representation could be a potential source of bias and compromise the external validity.

## 6 Future directions

In the findings of this systematic review, we underscore the urgent need for methodologically rigorous research to establish the potential role of acupuncture in the management of ODD. Given the pervasive risk of bias, heterogeneity, and low certainty of evidence identified across the included studies, future research should prioritize the design and execution of high-quality randomized controlled trials that adhere to CONSORT and STRICTA guidelines. Essential methodological improvements include adequate randomization and allocation concealment, appropriate sham controls, blinded outcome assessment, and complete reporting of adverse events. Furthermore, trials should incorporate standardized diagnostic criteria for each ocular condition, such as the DEWS II criteria for dry eye disease or the American Diabetes Association position statement for diabetic retinopathy, in order to ensure population homogeneity and comparability across studies.

Future research should focus in developing and validating standardized acupuncture protocols tailored to each inflammation-related ocular degeneration. Currently, the reviewed studies employed a highly heterogeneous point selections, stimulation parameters, and treatment regimens. Consequently, there is a missing replication and clinical implementation. A consensus-driven approach involving acupuncture experts and ophthalmologists could establish core acupoint prescriptions, adequate session frequency, and treatment duration for each condition, thereby facilitating the evidence synthesis through meta-analysis. Additionally, translational research integrating biomarker-based outcome measures, such as tear inflammatory cytokines or oxidative stress markers, help us to elucidate the mechanistic basis of acupuncture’s purported anti-inflammatory and antioxidant effects. Pragmatic trials embedded in routine clinical settings could also assess the feasibility, acceptability, and cost-effectiveness of integrating standardized acupuncture protocols as an adjunct to conventional ophthalmologic care. Ultimately, only through rigorous, transparent, and internationally collaborative research can we establish or refute the therapeutic value of acupuncture as a complementary treatment of inflammation-related ocular degenerations.

## 7 Conclusion

Current clinical evidence on the use of acupuncture in treating inflammation-related ocular degeneration disease remains limited, and the quality of the studies is very low, warranting a cautious interpretation of its therapeutic efficacy for clinical practice.

## Data Availability

The original contributions presented in the study are included in the article/supplementary material, further inquiries can be directed to the corresponding author.

## Appendix A1

### Excluded studies with reasons

**Table:**

ID	Reason
Dry eye syndrome	
Zhang Y, et al. (2007) (61)	Not full access
Gao WP, et al. (2010) (62)	Language
Jeoun JH, et al. (2010) (63)	Without control group
Wei LX, et al. (2010) (64)	Language, Moxibustion
Shi JL, et al. (2012) (65)	Language
Lin T, et al. (2015) (66)	Outcome
Xie W, et al. (2018) (67)	Language
Fu W, et al. (2018) (68)	Language
Jing L, et al. (2019) (69)	Language
Zhu D, et al. (2019) (70)	Language
Cheng J, et al. (2019) (71)	Language
Zhang D, et al. (2020) (72)	Pilot study
Zhang JW, et al. (2020) (73)	Language
Lee JH, et al. (2021) (74)	Pilot study
Hu WL, et al. (2021) (101)	Laser acupuncture
Sun YZ, et al. (2022) (75)	Language
Xhao L, et al. (2023) (76)	Language
Chia-yi L, et al. (2023) (77)	Acupoint steam-warming
Liu X, et al. (2023) (78)	Language
Shing, et al. (2024) (79)	Inadequate control group
Leedasawat P, et al. (2024) (80)	Massage
Diabetic retinopathy	
Zhang ZL, et al. (2006) (41)	Language
Chiu, et al. (2011) (30)	Outcome
Macular degeneration	
Krenn H (2008) (81)	Without control group
Jiao NJ (2011) (82)	Language
Li G, et al. (2017) (83)	Language
Yang W, et al. (2018) (84)	Language
Wu JG, et al. (2020) (85)	Case report
Yan Y, et al. (2021) (86)	Case report
Geng Z, et al. (2022) (87)	Case report
Usmani, B et al. (2023) (88)	Pilot study
Glaucoma	
Uhring S, et al. (2003) (89)	Without a control group, Language
Kurusu M, et al. (2005) (90)	Without a control group
Ewer H, et al. (2008) (91)	Without an adequate control group
Huo Q, et al. (2009) (92)	Language
Her JS, et al. (2010) (45)	Auricular therapy
Liu W, et al. (2011) (93)	Language
Xu H, et al. (2012) (94)	Case reports, language
Wang R (2017) (95)	Language
Valueva I. V, et al. (2018) (96)	Language
Wu AM, et al. (2019) (97)	Language
Liu J, et al. (2020) (98)	Language
Vanzini, et al. (2020) (99)	Laser acupuncture, without a control group
Ismail AMA, et al. (2021) (102)	Acupuncture + yoga
Papadopoulos G, et al. (2022) (100)	Case report
